# Analog series-based scaffolds: computational design and exploration of a new type of molecular scaffolds for medicinal chemistry

**DOI:** 10.4155/fsoa-2016-0058

**Published:** 2016-10-04

**Authors:** Dilyana Dimova, Dagmar Stumpfe, Ye Hu, Jürgen Bajorath

**Affiliations:** 1 Department of Life Science Informatics, B-IT, LIMES Program Unit Chemical Biology & Medicinal Chemistry, Rheinische Friedrich-Wilhelms-Universität, Dahlmannstr. 2, D-53113 Bonn, Germany

**Keywords:** analog series, analog series-based scaffold, framework, matched molecular pair, privileged substructure, scaffold

## Abstract

**Aim:**

Computational design of and systematic search for a new type of molecular scaffolds termed analog series-based scaffolds.

**Materials & methods:**

From currently available bioactive compounds, analog series were systematically extracted, key compounds identified and new scaffolds isolated from them.

**Results:**

Using our computational approach, more than 12,000 scaffolds were extracted from bioactive compounds.

**Conclusion:**

A new scaffold definition is introduced and a computational methodology developed to systematically identify such scaffolds, yielding a large freely available scaffold knowledge base.

## Lay abstract

In medicinal chemistry and drug design, so-called scaffolds are used to represent core structures of bioactive compounds. Over the past 20 years, a formal scaffold definition has predominantly been applied that considers molecules to consist of ring structures, which represent the scaffold, and chemical groups attached to rings. Herein, we introduce a new scaffold concept, which takes compound series and chemical reaction information into account.

## Graphical abstract

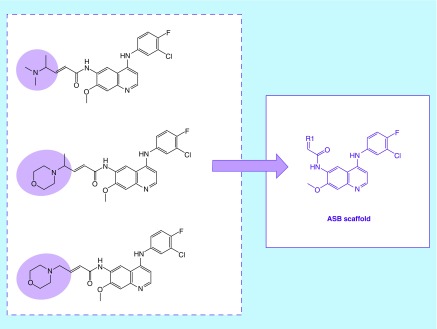

We introduce a new scaffold concept for medicinal chemistry. The figure illustrates how an ‘analog series-based scaffold’ is obtained from a series of structural analogs.

**Figure 1.**
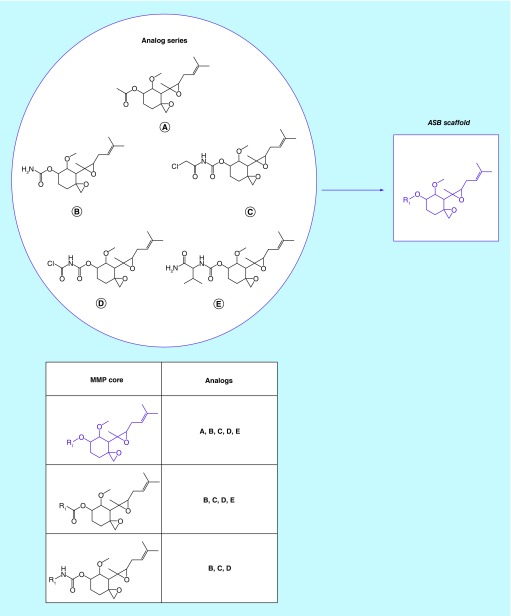
**Analog series-based scaffold identification.** For a small analog series consisting of five compounds, all possible matched molecular pair (MMP) cores are shown. The core shared by all analogs **(A–E)** represents the analog series-based scaffold (purple).

**Figure 2.**
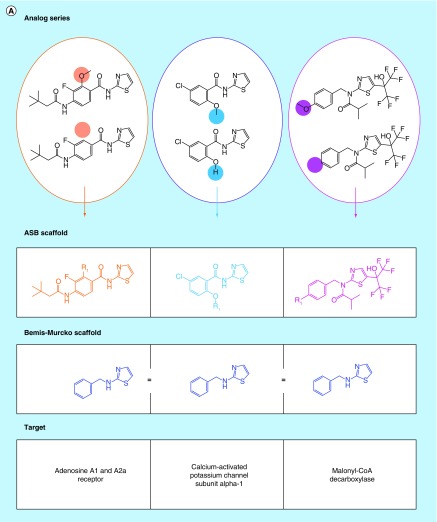

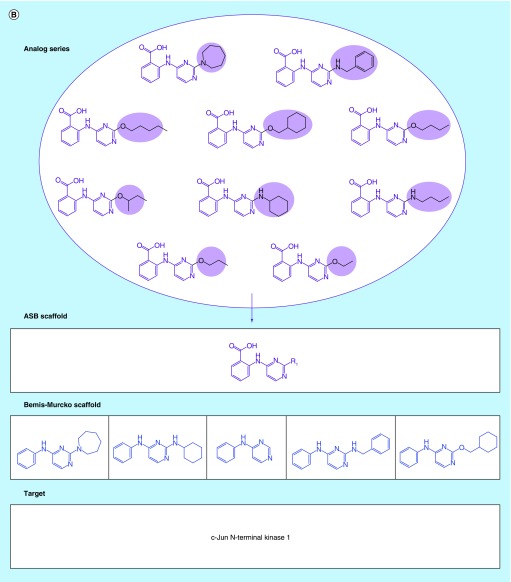
**Analog series-based scaffolds of exemplary analogs series.** **(A)** Analog series-based (ASB) scaffolds from three different analog pairs (smallest possible series) are shown and color-coded according to substitution sites in analogs. Targets of each analog pair are provided. All six compounds share the same Bemis–Murcko scaffold (blue) but each pair yields a different ASB scaffold. **(B)** For an exemplary analog series containing 10 c-Jun N-terminal kinase 1 inhibitors, the corresponding ASB scaffold (purple) and all Bemis–Murcko scaffolds of the analogs (blue) are shown. In this case, the analog series yields five distinct Bemis–Murcko scaffolds.

In medicinal and computational chemistry, the term scaffold is generally used to refer to core structures of compounds [1,2], which are also termed frameworks [2]. Of particular interest are scaffolds that represent active compounds and analog series [2], or are used as starting points for synthesis of analogs or chemical libraries [3]. Furthermore, the reduction of compounds to core structures makes it possible to structurally organize and classify large compound collections [4]. Moreover, a major attraction of the scaffold concept in medicinal chemistry is the association of core structure motifs with specific biological activities [2], which corresponds to the quest for privileged substructures [4,5], in other words, scaffolds representing compounds that are preferentially active against members of individual target families [5]. The underlying idea is that if a scaffold with privileged substructure character is identified it can be used as a template for target-directed compound or library design.

Although scaffolds are often assessed in a subjective manner through a chemist's eye, for a systematic evaluation of scaffolds and computational analysis, a generally applicable and consistent definition is required [2]. A first formal definition of scaffolds or frameworks was introduced by Bemis and Murcko in 1996 [6]. Compounds were considered to be composed of different components including ring systems, chemical linker fragments connecting rings, and substituents (R-groups) at rings and linkers. The scaffold of a compound was then defined to consist of all of its rings and linkers connecting them. Accordingly, a scaffold was obtained from a compound by removal of all substituents [6]. The Bemis–Murcko definition of scaffolds is not without intrinsic shortcomings from a chemistry perspective. By definition, scaffolds must contain ring structures and the addition of a ring to a compound always yields a new scaffold. This is not consistent with analog generation strategies where rings are often added to scaffolds as R-groups [2]. In addition, for example, chemical reaction information is not considered in scaffold generation. However, the Bemis–Murcko definition is generally applicable and provides a consistent basis for computational identification of scaffolds in compound datasets of any source. Consequently, although scaffolds can be rationalized in different ways, the Bemis–Murcko approach has dominated scaffold analysis in computational and medicinal chemistry over the past 20 years [1,2].

Herein, we present a conceptually distinct approach to generate scaffolds for medicinal chemistry applications and provide a large collection of new scaffolds.

## Methodological concept

The approach introduced herein focuses on a new way to define scaffolds and involves different steps. From the currently available universe of bioactive compounds, analog series are extracted with the aid of the matched molecular pair (MMP) formalism. An MMP is defined as a pair of compounds that are only differentiated by a chemical modification at a single site [7]. As such, an MMP consists of a common core, termed MMP core, and a pair of exchanged substituents. We note that the MMP core itself is not necessarily representing a scaffold because it may contain multiple shared substituents (i.e., the structural difference between MMP compounds is limited to one – and only one – site). Combining methods originating from our laboratory, MMPs are systematically generated from active compounds following retrosynthetic RECAP rules [8] yielding RECAP-MMPs [9]. Accordingly, bonds in compounds formed by predefined chemical reactions are systematically cleaved, which represents a retrosynthetic fragmentation scheme, and all possible MMPs are assembled. These RECAP-MMPs (in the following simply referred to as MMPs) are then organized in molecular networks in which nodes represent compounds and edges pairwise MMP relationships. Each disjoint network component (cluster) represents a distinct series of analogs [10]. We emphasize that the isolation of analog series as reported previously provides the basis for the design and generation of conceptually new scaffolds, which is the topic of our current study. From systematically identified analog series, new scaffolds are isolated. Furthermore, each series is searched for the presence of ‘structural key’ (SK) compounds that capture all MMP relationships present in a given analog series. In other words, an SK compound participates in the formation of MMPs with all other compounds within a series and is thus a central chemical entity representing the series. An SK compound yields one or more MMP cores that are shared with other analogs and can be used to generate all existing and additional analogs following chemical reaction rules. For scaffold design, an MMP core of an SK compound is strongly preferred that captures relationships with all analogs comprising a series.

Therefore, an MMP core of an SK compound covering structural relationships with all other analogs of a series is defined as an ‘analog series-based’ (ASB) scaffold.

This definition represents the central idea underlying our approach. If multiple qualifying cores exist, which is possible, the largest one (i.e., with the largest number of nonhydrogen atoms) is selected as an ASB scaffold.

Characteristic features of ASB scaffolds include that they are systematically derived from individual series of bioactive analogs, represent structural relationships between analogs and are consistent with chemical reaction information, are conceptually distinct from Bemis–Murcko scaffolds and other previously considered core structure definitions and are annotated with activity information because they are exclusively derived from series of active compounds.

Figure 1 schematically illustrates the computational identification of ASB scaffolds. From bioactive compounds, all analog series are isolated and for each series, SK compounds are identified. From each SK compound, all MMP cores are derived. A core representing all analog relationships within a series principally qualifies as an ASB scaffold.

## Materials & supplementary methods

### Compounds & activity data

Bioactive compounds were assembled from version 21 of ChEMBL [11], the major public repository of compounds and activity data from medicinal chemistry sources. The following selection criteria were applied to select compounds for which high-confidence activity data were available. First, only compounds involved in direct interactions (target relationship type ‘D’) with human targets at the highest confidence level (target confidence score 9) were taken. Second, two different types of potency measurements were considered including assay-independent equilibrium constants (K_i_ values) and assay-dependent IC_50_ values. Approximate measurements associated with ‘>’, ‘<’ or ‘∼’ were discarded. If a compound had multiple K_i_ or IC_50_ values for the same target, the geometric mean of the values was calculated as the final potency annotation provided that all values fell into the same order of magnitude. Otherwise, the values were discarded. Applying these selection criteria, a total of 167,290 unique compounds were obtained with activity against a total of 1594 targets.

### RECAP-MMPs

For the pool of 167,290 bioactive compounds, RECAP-MMPs were systematically generated. Previously established fragment size restrictions were applied to limit MMPs to pairs of compounds consisting of typical analogs, in other words, compounds distinguished by relatively small substituents [12]. Therefore, the size of the conserved MMP core was required to be at least twice the size of the larger substituent, which was permitted to consist of at most 13 heavy atoms. These restrictions ensured that substituents were limited in size to maximally a condensed two-ring system with no more than three additional atoms. These MMPs were then used to identify analog series, SK compounds and ASB scaffolds, as presented in the following.

### Implementation

The ASB scaffold method and routines for compound retrieval and activity data mining were implemented using in-house Perl and Python scripts with the aid of KNIME [13] protocols and the OpenEye chemistry toolkit [14].

## Results & discussion

Our implementation of the ASB scaffold methodology as described above was used to search the large pool of selected bioactive compounds for qualifying scaffolds.

### Analog series & SK compounds

The selected compounds yielded a total of 17,371 unique analog series that were determined following a previously reported three-step procedure [10], as discussed above. For 14,988 series (86%), SK compounds were identified that formed MMP relationships with all other analogs within a series. For each SK compound, all MMP cores were derived. In 12,294 of these series (71%), one or more MMP cores were found representing structural relationships with all other analogs. In these instances, each analog within a series formed MMP relationships with all others and thus qualified as an SK compound, which also applies to the example shown in Figure 1. Analog series and SK compound statistics are provided in Table 1.

### ASB scaffold distribution

Each of the 12,294 analog series with qualifying MMP cores yielded a unique ASB scaffold. Thus, ASB scaffolds were successfully identified in 71% of all analog series isolated from bioactive compounds, forming a large pool of newly derived scaffolds. As reported in Table 1, these scaffolds represented compounds active against a total of 1184 targets. ASB scaffolds included 6986 entities associated with single and 5308 entities associated with multitarget activity. The former subset of nearly 7000 new scaffolds also is a prime knowledge base for revisiting the search for privileged substructures.

For the remaining 2694 analog series with SK compounds (Table 1), in which a single MMP core was not shared by all analogs, two to nine MMP cores from SK compound(s) covered all analog relationships.

### Scaffold relationships

Figure 2 reveals different relationships between ASB scaffolds and standard Bemis–Murcko scaffolds. In Figure 2A, three pairs of analogs are shown that represent different series. All of these compounds contain the same Bemis–Murcko scaffold but for each series with activity against different targets, a distinct ASB scaffold is obtained. By contrast, analogs comprising the series in Figure 2B (with activity against a single target) yield five different Bemis–Murcko scaffolds but only one ASB scaffold representing the entire series, which is chemically intuitive and advantageous for medicinal chemistry applications.

## Conclusion & future perspective

Scaffolds are intensely explored in medicinal chemistry computer-aided drug design. For computational analysis, a consistent and generally applicable scaffold definition is essential. In this work we have introduced a conceptually new way to define scaffolds and a computational methodology to search for these scaffolds. As defined herein, ASB scaffolds are analog series-centric in nature, comprehensively capture structural relationships, conform to retrosynthetic rules, and are annotated with biological activities. The introduction of ASB scaffolds was in part motivated by attempting to further increase the relevance of generalized scaffolds for the practice of medicinal chemistry. ASB scaffolds were successfully obtained from the majority of currently available analog series, hence indicating the relevance of the underlying concept and robustness of its implementation. Going forward, further extensions of the ASB scaffold approach can be considered. So far a single MMP core of an SK compound has been selected as an ASB scaffold, but it would be readily possible to select multiple qualifying cores for a series, if available, for example, the smallest and largest one. This would further increase the number of ASB scaffolds for consideration. Moreover, in cases where no single qualifying MMP core is available but multiple cores capturing subsets of analog relationships – as observed for more than 2000 series in our analysis – it would be possible to combine structural information from these cores, for example, by calculating their maximum common substructure. This transformation might cause an at least partial loss of implicit reaction information, but so derived ‘consensus’ scaffolds might nonetheless be useful for compound mapping or design. It is of course also possible to further increase reaction information associated with ASB scaffolds by adding additional retrosynthetic rules to the MMP generation step. Hence, various opportunities exist to further extend the ASB scaffold methodology for specific applications.

As a part of this study, the large pool of more than 12,000 ASB scaffolds reported herein and associated activity information are made freely available as an open access deposition [15] under the authors’ names. It is hoped that these scaffolds are interesting and useful for medicinal chemistry applications and that their availability might trigger further research in the area of molecular scaffolds and privileged substructures.

**Table 1.** **Analog series, structural key compounds and analog series-based scaffolds.**

	**All analog series**	**Analog series with SK CPDs**	**Analog series with ASB scaffold**
Analog series (n)	17,371	14,988 (86%)	12,294 (71%)
Single target	9171	8273	6986
Multiple targets	8200	6715	5308
CPDs (n)	96,889	57,757 (60%)	39,467 (40%)
SK CPDs (n)	–	42,894 (44%)	39,467 (40%)
Analog series size	2–336	2–46	2–44
Mean	5.6	3.9	3.2
Targets (n)	1382	1268	1184

The global distribution of analog series obtained from selected ChEMBL compounds (CPDs) is reported together with compound and target numbers. Corresponding statistics are provided for analog series containing at least one SK compound and series yielding an ASB scaffold.

ASB: Analog series-based; CPD: Compound; SK: Structural key.

**Executive summary**

**Methodological concept**

With analog series-based (ASB) scaffolds, a novel scaffold definition has been introduced.
ASB scaffolds are derived from analog series, comprehensively capture structural relationships between analogs and contain synthetic information.

**ASB scaffold distribution**

From more than 70% of analog series extracted from bioactive compounds, ASB scaffolds were obtained.
A large pool of more than 12,000 ASB scaffolds with broad coverage of more than 1000 targets has been assembled.
By design ASB scaffolds are annotated with activity information and hence enable revisiting the privileged substructure concept.

**Conclusion & future perspective**

ASB scaffolds are made freely available for medicinal chemistry and chemical informatics applications.
